# The Evolution of Gastrointestinal Bleeding: A Holistic Investigation of Global Outputs with Bibliometric Analysis

**DOI:** 10.5152/tjg.2022.22007

**Published:** 2022-12-01

**Authors:** Emre Kudu, Faruk Danış

**Affiliations:** 1 Department of Emergency Medicine, Marmara University Pendik Education and Research Hospital, İstanbul, Turkey; 2 Department of Emergency Medicine, Bolu İzzet Baysal Training and Research Hospital, Bolu, Turkey

**Keywords:** Bibliometric analysis, gastrointestinal bleeding, gastrointestinal hemorrhage, trends

## Abstract

**Background:**

Gastrointestinal bleeding is one of the main presentations in emergency department admissions. Although there has been much improvement in prevention, diagnosis, and treatment recently, patients with GIB still have high morbidity and mortality. This study aimed to analyze the scientific articles on gastrointestinal bleeding published between 1980 and 2020 using statistical and bibliometric methods.

**Methods:**

Articles about gastrointestinal bleeding published between 1980 and 2020 were downloaded using the Web of Science database and analyzed using statistical methods. Network visualization maps were used to identify trending topics. Correlation studies were evaluated using Spearman’s correlation coefficient. Nonlinear regression analysis (exponential model) was used to estimate the number of articles in future years.

**Results:**

A total of 12 568 publications about gastrointestinal bleeding were found. Forty percent (n = 5033) of these publications were articles. The top 3 contributing countries to the literature were the United States of America (1646, 32.7%), the United Kingdom (433, 9%), and Germany (391, 7.7%). The top three journals with the most publications were *Gastrointestinal Endoscopy* (172), *American Journal of Gastroenterology* (165), and *Digestive Diseases and Sciences* (161). The effect of countries’ gross domestic product levels on article productivity on gastrointestinal bleeding was significant (*r* = 0. 770, *P* < .001).

**Conclusion:**

In this comprehensive study, a summary of 5033 articles was presented. We think that these detailed analyses will be a quick source to show the past, present, and future of this subject to those who are currently working on gastrointestinal bleeding.

**Main Points**

- According to our analysis in the Web of Science database, there are more than 12 568 publications in a 40-year period and 5033 of them are articles, and this number is predicted to increase exponentially.
- Most of these studies have been done by gastroenterology and hepatology departments, but according to trend analysis, departments including treatment areas such as radiology and nuclear medicine have made a splash recently, and more studies are expected on these issues in the near future.
- Publication productivity of countries is correlated with gross domestic product (GDP), and high GDP directly increases productivity.
- Although epidemiological studies have received a lot of citations in the long-term, it is seen that new studies examining new treatment modalities or patient outcomes receive a lot of citations in a short time.

## Introduction

Gastrointestinal bleeding (GIB) is one of the main presentations in emergency department admissions. It has high mortality and morbidity rates and requires significant resources. Gastrointestinal bleeding is divided into 2 categories according to its location; bleeding proximal to the ligament of Treitz is called upper gastrointestinal bleeding (UGIB) and bleeding from below is called lower gastrointestinal bleeding (LGIB). Both have unique etiologies and UGIB is more common.

Upper gastrointestinal bleeding has an incidence of 50-100/100 000 and the mortality ranges between 3% and 14%.^1,2^ The incidence of LGIB is 33-87/100 000. Mortality rates of LGIB are estimated to be between 2% and 4%.^3,4^ Both are more common in men and their incidence and mortality rates increase with age.

The approach to GIB cases first starts with the stabilization of the patient. After initial resuscitation, identifying the source of bleeding is a clinical priority and distinguishing between upper and lower GIB can be challenging. Clinical features such as age and sex, comorbidities, medications, previous bleeding history, and type and duration of bleeding can help in the diagnosis.^5^ Lower and/or upper endoscopy are usually the first tests performed and their use has recently started to increase.^6,7^ These interventions help in treatment as well as in diagnosis.^7^ Gastrointestinal bleeding is managed by many clinicians such as emergency medicine physicians, internal specialists, gastroenterologists, surgeons, radiologists, and hematologists because it concerns all these procedures and approaches.

Bibliometry is the analysis of scientific articles and other scientific publications on a specific subject using statistical methods.^8^ The number of publications in the literature is constantly increasing and the amount of medical information doubles every 5 years on average.^9^ Considering this increasing amount of information and the limited time available to researchers, bibliometric studies are of great help in making a literature review in a short time and approaching the subject with holistic dominance. In parallel with this information, studies based on statistical and bibliometric analyses have been conducted on many important medical subjects recently.^10-13^ Bibliometric research can identify the most active authors, institutions, journals, the most cited influential studies, and co-citations on a subject and reveal international collaborations.^14^

Although the number of global studies about GIB has increased in recent years, there is still no bibliometric study in the literature about this important subject. In this study, it was aimed to analyze scientific articles on GIB published between 1980 and 2020 using statistical and bibliometric methods.

## Materials and Methods

The Web of Science (WoS) database (by Clarivate Analytics) was used for the literature review. We used gastrointestinal bleeding (GI bleed, GI bleeding, etc.) and gastrointestinal hemorrhage (hemorrhages, haemorrhage, etc.) as keywords in the WoS search. In order not to include studies that were not directly related to the subject, publications were searched only in the title section of the studies. With this search method, all articles with gastrointestinal bleeding (e.g., GI bleeding, bleed) and gastrointestinal hemorrhage (e.g., hemorrhages, haemorrhage) in the title section were obtained and these articles were downloaded from the WoS database. Studies that were labeled in the field of pediatric research or that included keywords such as child, children, and pediatrics in the title, abstract, or keywords section were excluded. The time interval was determined as 1980-2020 (Access date: June 15, 2021). Reproducibility codes for researchers to access similar documents (search findings may vary depending on different access dates) were as follows: (Title: “gastrointestinal bleed*” OR Title: “GI bleed*” OR Title: “gastrointestinal (GI) bleed*” OR Title: “gastrointestinal hemorrhage*” OR Title: “gastrointestinal haemorrhage*” OR Title: “GI hemorrhage*” OR Title: “GI haemorrhage*” OR Title: “gastrointestinal (GI) hemorrhage*” OR Title: “gastrointestinal (GI) haemorrhage*” NOT Topic: (child*) NOT Topic: (pediatric*) Refined by: [excluding] Web of Science Categories: (Pediatrics) Timespan: 1980-2020. Indexes: SCI-Expanded, SSCI, A&HCI, CPCI-S, CPCI-SSH, BKCI-S, BKCI-SSH, ESCI.

### Statistical Analysis

Statistical analyses were performed using the Statistical Package for the Social Sciences software (Version 22.0, IBM Corp.; Armonk, NY, USA) package program. Kolmogorov–Smirnov tests were used to analyze the normality distribution of the variables. To determine whether the economic power of the countries affected the productivity of world publications on GIB, the difference between the number of articles produced by countries of the world and some economic development indicators [gross domestic product (GDP) and gross domestic product per capita (GDP per capita)] of the countries was calculated using data obtained from the World Bank. Spearman’s correlation coefficient was used in accordance with the data distribution to determine the correlations.^15^ Visually, this relationship was evaluated with scatterplots between the logarithms of GDP and GDP per capita values and the number of published articles by countries. Nonlinear regression analysis (exponential model) was used to predict the number of publications in the coming years. The *R* ^2^ value was used to evaluate model success in regression analysis. The limit of statistical significance was accepted as *P* < .05. The VOSviewer package program (Version 1.6.16, Leiden University’s Centre for Science and Technology Studies) was used for bibliometric network visualizations.^16^ The website “https://app.datawrapper.de” was used for world map drawing.

## Results

As a result of the literature search, a total of 12 568 publications about GIB were found in the WoS database between 1980 and 2020. When we looked at the distribution of these publications, 40% (n = 5033) were articles, 37.8% (n = 4757) were meeting abstracts, 7.9% (n = 995) were editorial material, 7.7% (n = 972) were letters, 4% (n =5 15) were reviews, 1.6% (n=204) were proceedings papers, and the remainder was other publication types (notes, corrections, book chapters, news items, discussions, early access, books, correction additions, data papers, retracted publications, and retractions). Bibliometric analyses were conducted on 5033 articles. Eighty-nine percent (n = 4489) of these articles were published in English, 5% percent (n = 254) in German, 2% (n = 118) in Spanish, 2% (n = 104) in French, and the remainder (n = 68) in other languages (Russian, Turkish, Hungarian, Japanese, Serbian, Greek, Icelandic, Polish, and Portuguese).

### Development and Future Trend of Publications

**Figure 1.**
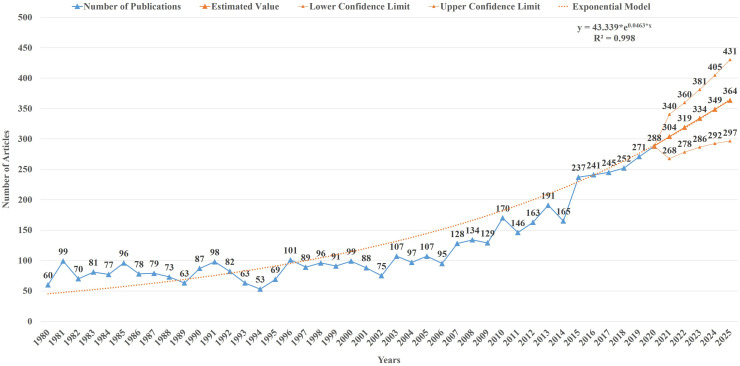
Number of articles published on gastrointestinal bleeding by years and estimation of the number of articles that will be published in the future.

The distribution of the number of published articles by years is shown in Figure 1 with a line graph. The nonlinear regression analysis results used to estimate the number of articles that could be published in 2021 and beyond are also shown in Figure 1. The compatibility of the exponential model with the data (*R* ^2^ = 0.9951) was quite high at 99.5%. According to the exponential model results, it is estimated that 304 (95% CI: 268-340) articles will be published in 2021 and 364 (95% CI: 297-431) articles will be published in 2025 (Figure 1).

### Active Research Areas

The top 10 research areas where articles published on GIB were most labeled were gastroenterology hepatology (n = 2173, 43.1%), medicine general internal (959, 19%), surgery (858, 17%), radiology nuclear medicine medical imaging (388, 7.7%), pharmacology pharmacy (199, 3.9%), cardiac cardiovascular systems (134, 2.6%), emergency medicine (122, 2.4%), medicine research experimental (104, 2%), peripheral vascular disease (94, 1.8%), and critical care medicine (79, 1.5%).

### Active Countries

**Figure 2.**
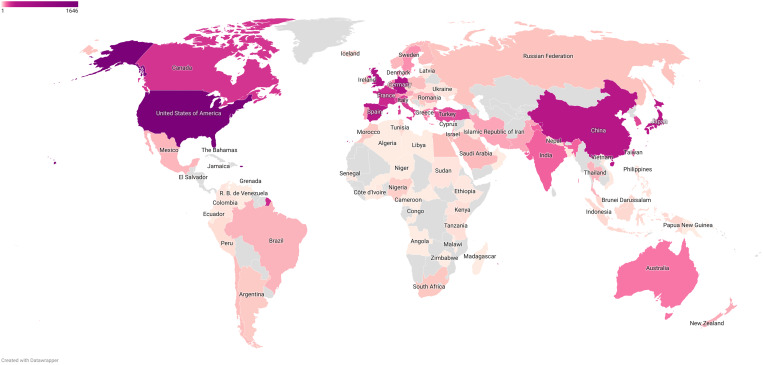
World map for the distribution of articles by country on gastrointestinal bleeding. In the indicator given at the top left of the figure, productivity increases from light to dark.

**Figure 3:**
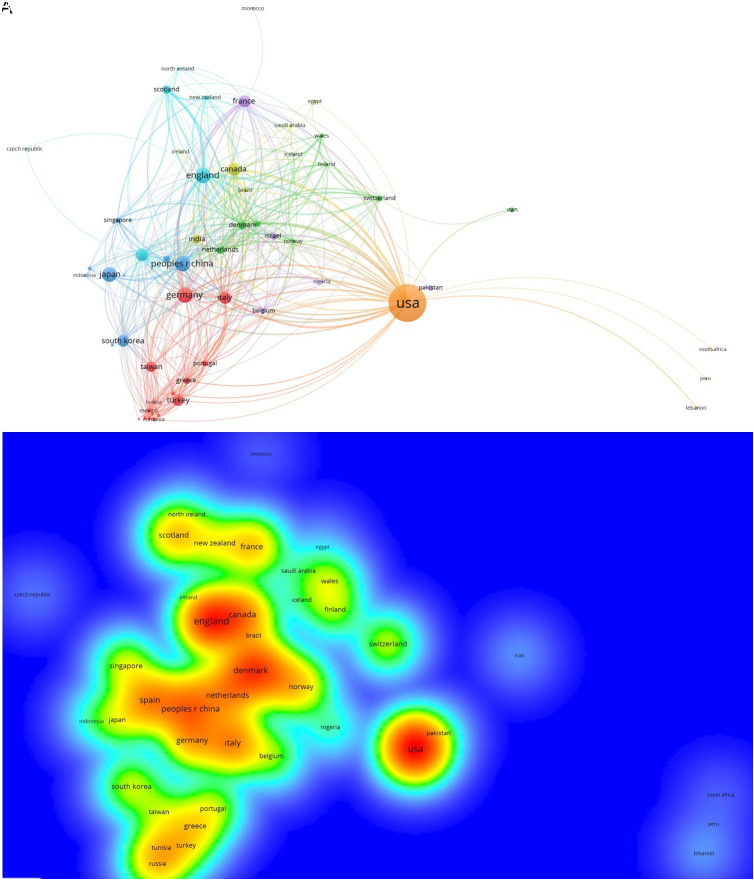
(A) Cluster analysis of international collaboration of worldwide countries on gastrointestinal bleeding. The size of the circle shows a large number of articles. The thickness of the lines indicates the strength of international collaboration. (B) Density map for international collaboration of worldwide countries on gastrointestinal bleeding. International collaboration increases from blue to red (blue-green-yellow-red).

The distribution of the number of articles by country is shown in Figure 2. The top 20 countries with the most articles published were the United States of America (1646, 32.7%), the United Kingdom (433, 9%), Germany (391, 7.7%), China (278, 5.5%), Japan (263, 5.2%), Spain (249, 4.9%), France (209, 4.1%), Canada (192, 3.8%), Italy (178, 3.5%), South Korea (158, 3.1%), Turkey (150, 2.9%), Taiwan (136, 2.7%), India (117, 2.3%), Australia (86, 1.7%), Denmark (85, 1.6%), the Netherlands (76, 1.5%), Switzerland (69, 1.3%), Israel (66, 1.3%), Greece (63, 1.2%), and Sweden (54, 1%). Cluster analysis was conducted among 55 countries that produced at least 5 articles from 115 countries producing publications about GIB and whose authors had international cooperation. According to the results of the analysis, 7 different clusters related to international cooperation were formed [cluster 1 (red color): 15 countries; cluster 2 (green color): 9 countries; cluster 3 (blue color): 9 countries; cluster 4 (yellow color): 6 countries; cluster 5 (purple color): 6 countries; cluster 6 (turquoise color): 6 countries; cluster 7 (orange color): 4 countries; Figure 3a]. The cooperation density map that was created according to the total cooperation scores calculated for the countries (the top 10 countries with the highest cooperation scores were the United States, the United Kingdom, Denmark, Italy, Canada, Spain, China, the Netherlands, Germany, and France, respectively) is presented in Figure 3b.

### Correlation Analysis

**Figure 4.**
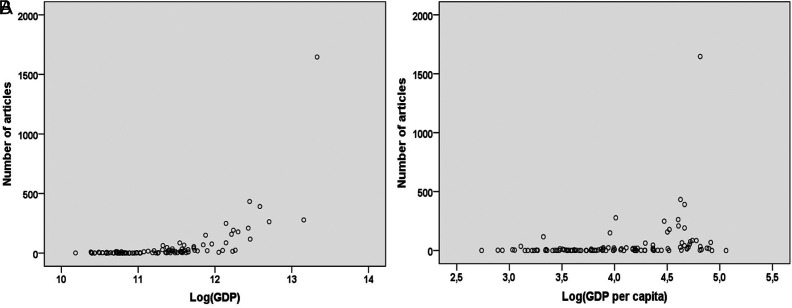
Scatterplot showing the relationships between the logarithms of countries’ gross domestic product (GDP), GDP per capita values, and their article productivity.

There was a high level of statistically significant positive correlations between the number of articles produced by countries on GIB with GDP and GDP per capita (*r* = 0.770, *P *< .001; *r* = .513, *P* < .001). The relationships between the logarithm of the GDP and GDP per capita values of the countries and the number of articles of the countries are visually shown in Figure 4 with a scatterplot.

### Active Authors

The top 10 authors who published most of the articles on GIB were Rockey DC (n = 31), Lanas A (n = 29), Jairath V (n = 25), Sung JJY (n = 23), Barkun AN (n = 21), Stanley AJ (n = 21), Rodriguez LAG (n = 20), Gralnek IM (n = 19), Saltzman JR (n = 19), and Wilcox CM (n = 19), respectively.

### Active Institutions

The top 10 most active universities that produced the most articles on GIB were Harvard University (n = 61), Mayo Clinic (n = 55), University of California Los Angeles (n = 4 9), McGill University (n = 46), University of Texas (n = 44), Chinese University Hong Kong (n = 43), Duke University (n = 43), University Hospital (n = 39), Veterans Admin Medical CTR (n = 35), and McMaster University (n = 34), respectively.

### Active Journals

**Figure 5.**
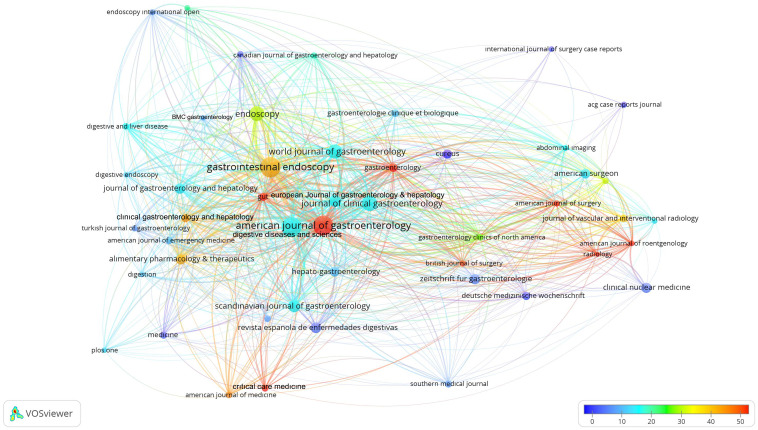
Network visualization map for citation analysis of active journals on gastrointestinal bleeding. The size of the circle shows a large number of articles. The number of citations from blue to red (blue-green-yellow-red) increases.

Five thousand thirty-three articles on GIB were published in 978 different journals. The first 48 most active journals producing 20 or more articles, the total number of citations by journal, and the average number of citations per article are presented in Table 1. The citation network visualization map created according to the average number of citations per article among these journals is presented in Figure 5.

### Citation Analysis

The top 20 most cited articles among the 5033 articles on GIB published between 1980 and 2020 are presented in Table 2. ^17-36^ In the last column of Table 2, the average number of citations per year is given.

### Co-citation Analysis

A total of 47 442 citations were made in the reference section of the 5033 articles analyzed. Among these studies, the first 8 studies that received the most co-citations (more than 150 citations) were Rockall et al^17^ (number of co-citations (NC): 387), Rockall et al^21^ (NC: 285), Blatchford et al^24^ (NC: 274), Barkun et al^37^ (NC: 256), Longstreth^33^ (NC: 181), Forrest et al^38^ (NC: 176), van Leerdam et al^36^ (NC: 174), and Longstreth^29^ (NC: 173), respectively.

### Trend Topics

**Figure 6.**
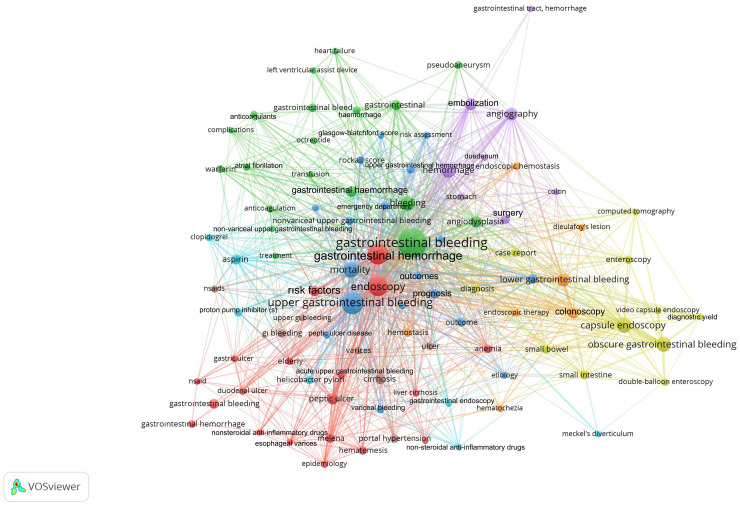
Network visualization map for cluster analysis based on keyword analysis on gastrointestinal bleeding. Colors show clustering. Keywords in the same cluster are of the same color. The circle size increases with the number of times the keyword is used.

**Figure 7.**
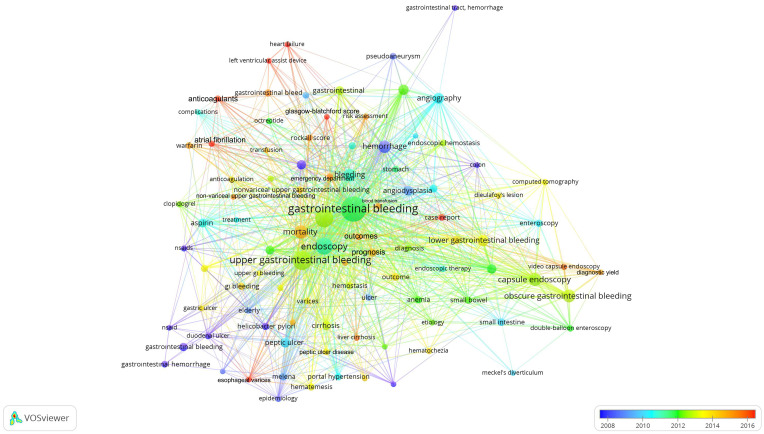
Network visualization map for trends on gastrointestinal bleeding. Indicator shows trend keywords from blue to red (blue-green-yellow-red). The circle size increases with the number of times the keyword is used.

Four thousand three hundred forty-one different keywords were used in all of the 5033 articles published on GIB. Among these keywords, 92 different keywords that were used in at least 15 different articles are shown in Table 3. The network visualization map of the clustering analysis results between these keywords is shown in Figure 6 [cluster 1 (red color): 19 keywords; cluster 2 (green color): 19 keywords; cluster 3 (blue color): 18 keywords; cluster 4 (yellow color): 11 keywords; cluster 5 (purple color): 8 keywords; cluster 6 (turquoise color): 7 keywords; cluster 7 (orange color): 7 keywords; cluster 8 (brown color): 7 keywords]. The trend network visualization map performed to reveal the trending topics is shown in Figure 7. Figure interpretations are explained to in legend.

## Discussion

When the number of articles published on GIB was evaluated by years, initially, it was 85 (53-107) articles/year between 1980 and 2006. This number increased to 154 (128-191) articles/year between 2007 and 2014 and 256 (237-288) articles/year between 2015 and 2020. In 2020, 288 articles were published and it was determined that there was an exponentially increasing trend in the number of articles in recent years. According to the results of nonlinear regression analysis, the number of articles will continue with an increasing exponential trend.

When we looked at the distribution of active research areas on GIB, gastroenterology and hepatology departments were leading with 43.1%. Later, we can say that departments that were mostly related to treatment parts such as surgery, radiology, nuclear medicine, medical imaging, and pharmacology came to the fore. Emergency medicine had a low level of 2.4%. Although almost all patients with GIB first present to the emergency department, this may be because treatment protocols require a multidisciplinary approach, and long-term follow-up of patients is completed by other departments. Another reason for this may be that the emergency department is newer compared with other departments such as internal medicine. Considering that the emergency department offers many resources and data for studies on this subject, we can say that will make up a larger portion of the whole with time.

Looking at the distribution of publications in terms of production of GIB articles in the most active countries, 17 of the first 20 countries that were most active in article production on GIB were developed countries; the other 3 countries (China, Turkey, and India) were developing countries. Although these 3 countries were developing, each had a large economy. In addition, when the results of the correlation analysis were examined in our study, a significant correlation was found between article production and GDP, which is one of the indicators of economic development. Similar to the bibliometric studies conducted on many different medical subjects in the literature, it has been observed that economic power is effective in publication production.^39^ A moderately significant correlation was determined with GDP per capita. When the scatter plots were examined, there was a higher correlation between article productivity and GDP rather than GDP per capita. The reason for this is that although some countries [Luxembourg (n = 0), United Arab Emirates (n = 3), Qatar (n = 4), Iceland (n = 9), and Hong Kong (n = 14)] have high GDP per capita, they do not have scientific article productivity. This shows that in addition to the economic power of the countries, other development indicators can also be effective in the production of articles. When the co-authorship cooperation of countries on GIB was examined in the density map, which was created according to the total cooperation scores between countries, the fact that countries with geographic neighbors (China–Japan–South Korea–Singapore), (France––Belgium), (Denmark–the Netherlands–Switzerland), (Italy–Turkey–Greece–Romania–Austria) are in the same cluster shows that geographic proximity has a significant effect on article production.

The journals that published the most articles on GIB were *Gastrointestinal Endoscopy*, *American Journal of Gastroenterology*, *Digestive Diseases and Sciences*, *Journal of Clinical Gastroenterology*, and *Endoscopy*, respectively. When the citation analyses of the journals were evaluated, the most effective journals according to the average number of citations per article published were *Gastroenterology*, *Gut*, *American Journal of Gastroenterology*, *Radiology*, and *American Journal of Roentgenology*, respectively. It is noteworthy that the journals with the most publications on GIB are not the same as those with the most citations. For example, although the *American Journal of Roentgenology* has 23 publications, it has a high citation count of 54.4 per publication. It is recommended that people who want to publish on this subject choose their journals by considering these 2 categories.

When the analyzed articles were evaluated according to the total number of citations they received, the most cited study was published in *Gut* by Rockall et al^17^ on “Risk assessment after acute upper gastrointestinal haemorrhage” in 1996. The aim of this study was to reveal the relative importance of risk factors for mortality after acute UGIB and to formulate a simple numerical scoring system that categorized patients according to risk. This study included 4185 patients, and as a result, age, shock status, comorbidities, diagnosis, major recent bleeding, and rebleeding were all individual mortality indicators.^17^ In addition, the fifth most effective study was found as the “Incidence of and mortality from acute upper gastrointestinal haemorrhage in the UK (1995),” and this article was also written by Rockall et al.^21^

The second most cited study was Villanueva et al’s^18^ “Transfusion strategies for acute upper gastrointestinal bleeding,” which was published in the *New England Journal of Medicine* in 2013. This study compared the liberal transfusion strategy with the restrictive transfusion strategy in terms of efficacy and safety, and as a result, it was seen that the restrictive transfusion strategy improved the outcomes in acute UGIB.

The third most cited study was “Risk of upper gastrointestinal bleeding and perforation associated with individual nonsteroidal anti-inflammatory drugs” by Rodriguez et al^19^ and was published in *The Lancet* in 1994. In this study, non-steroidal anti-inflammatory drugs (NSAIDs) and other factors that increase the risk of UGIB were examined, and it was emphasized that NSAIDs should be used carefully in people with an increased risk of GIB such as older patients, smokers, and those with a history of peptic ulcers.^19^

When the studies were evaluated according to the average number of citations per year, the most efficient article was Villanueve et al’s^18^ study in 2013. The second most influential article was Pennazio et al’s^20^ “Outcome of patients with obscure gastrointestinal bleeding after capsule endoscopy: Report of 100 consecutive cases” in 2004. The third most influential study was Rockall et al’s^17^ article “Risk assessment following acute gastrointestinal haemorrhage.” The fourth most influential study was Abraham et al’s^40^ article “Comparative risk of gastrointestinal bleeding with dabigatran, rivaroxaban, and warfarin: A population-based cohort study,” published in the *British Medical Journal* in 2015. When the citations were examined carefully, it was seen that epidemiologic studies received excessive citations in the long term. It was observed that topics that affected the outcome of patients, shaped treatment algorithms, examined the effectiveness and complications of endoscopy, examined new-generation anticoagulants, were up-to-date, and attracted great attention by achieving many citations in a short time.

According to the co-citation numbers of the 5033 articles analyzed, the most influential studies are Rockall et al,^17^ Rockall et al^21^ Blatchford et al^24^ Barkun et al,^37^ Longstreth,^33^ Forrest et al,^38^ van Leerdam,^36^ Longstreth,^29^ respectively. We can suggest that physicians and researchers who want to publish on the subject of GIB should first examine these publications. These will be of great help in identifying tricks or areas for improvement in their work/publication that should be carefully approached.

Clusters of keywords were analyzed to identify trending topics. It was determined that the trend topics studied in recent years were atrial fibrillation, esophageal varices, anticoagulants, heart failure, left ventricular assist devices, Glasgow–Blatchford scores, video capsule endoscopy, liver cirrhosis, warfarin, risk assessment, and Rockall scores. The most mentioned keywords were aspirin, NSAIDs, clopidogrel, hemostasis, epidemiology, heart failure, treatment, left ventricular assist device, risk assessment, nonsteroidal anti inflammatory drugs, gastrointestinal endoscopy, outcomes, and double-balloon enteroscopy. In parallel with the citation analysis, keyword analyses also showed that epidemiology, drug interactions, and new treatment modalities were hot topics.

When we examined the literature in detail, no previous bibliometric study about GIB was found. In our study, all of the scientific articles published on GIB between 1980 and 2020 were analyzed comprehensively. To the best of our knowledge, our study is the first detailed bibliometric research on this subject. The presence of keyword analysis, trend topic analysis, cluster analysis, correlation, and regression analyses in our study, apart from citation analysis, is another superior aspect of our study.

To mention the limitations of our study, only the WoS database was used. Our analyses do not include other databases such as Scopus, PubMed, and Google Scholar. While making this choice, the WoS database’s features such as having a wide network of journals, already containing many important studies from PubMed, Scopus, and Google Scholar, and providing enough data to allow for many analyses such as citation and co-citation analysis were taken into account.^41^ When we looked at the literature, it was seen that the WoS was used in most bibliographic studies.^10-13^

We shared a summary of 5033 articles published between 1980 and 2020 in this wide bibliometric study on GIB, which has had an increasing trend in the number of articles published in recent years. We think that these detailed analyses will be a guide for people who are interested in this subject. In addition, it will be a quick resource to show the past, present, and future of this subject to those who are currently working on GIB. It will also help those planning new studies to see which topics are trending, which topics are currently prominent, and which topics need to be studied.

## Figures and Tables

**Table 1.** Forty-Eight Most Active Journals with More Than 20 Articles on Gastrointestinal Bleeding

**Journals**	**RC**	**C**	**AC**	**Journals**	**RC**	**C**	**AC**
*Gastrointestinal Endoscopy*	172	7005	40.7	*Southern Medical Journal*	30	249	8.3
*American Journal of Gastroenterology*	165	9599	58.2	*American Journal of Medicine*	28	1245	44.5
*Digestive Diseases and Sciences*	161	2683	16.7	*Critical Care Medicine*	28	1508	53.9
*Journal of Clinical Gastroenterology*	105	1690	16.1	*Digestive and Liver Disease*	28	469	16.8
*Endoscopy*	100	3002	30.0	*Postgraduate Medical Journal*	28	236	8.4
*World Journal of Gastroenterology*	98	1658	16.9	*Gastroenterology Clinics of North America*	27	730	27.0
*Scandinavian Journal of Gastroenterology*	76	1200	15.8	*American Journal of Surgery*	26	1329	51.1
*European Journal of Gastroenterology and Hepatology*	72	1148	15.9	*British Journal of Surgery*	26	1252	48.2
*Revista Espanola De Enfermedades Digestivas*	60	290	4.8	*Diseases of the Colon and Rectum*	26	806	31.0
*Clinical Nuclear Medicine*	57	316	5.5	*Endoscopy International Open*	26	210	8.1
*Alimentary Pharmacology and Therapeutics*	56	2269	40.5	*Turkish Journal of Gastroenterology*	26	202	7.8
*Zeitschrift Fur Gastroenterologie*	55	370	6.7	*ACG Case Reports Journal*	25	37	1.5
*Journal of Gastroenterology and Hepatology*	54	792	14.7	*Canadian Journal of Gastroenterology and Hepatology*	25	472	18.9
*American Surgeon*	52	732	14.1	*Gastroenterology Research and Practice*	25	100	4.0
*Hepato-Gastroenterology*	51	550	10.8	*Abdominal Imaging*	24	402	16.8
*Cureus*	47	31	0.7	*American Journal of Roentgenology*	23	1251	54.4
*Gastroenterology*	42	4386	104.4	*BMC Gastroenterology*	23	219	9.5
*Clinical Gastroenterology and Hepatology*	40	1701	42.5	*Digestion*	23	260	11.3
*Gut*	40	3596	89.9	*Digestive Endoscopy*	23	275	12.0
*Deutsche Medizinische Wochenschrift*	38	102	2.7	*Cardiovascular and Interventional Radiology*	22	326	14.8
*Journal of Vascular and Interventional Radiology*	36	1368	38.0	*Plos One*	22	281	12.8
*Gastroenterologie Clinique Et Biologique*	34	387	11.4	*Surgical Endoscopy and Other Interventional Techniques*	21	439	20.9
*Medicine*	32	126	3.9	*International Journal of Surgery Case Reports*	21	66	3.1
*American Journal of Emergency Medicine*	30	330	11.0	*Radiology*	20	1122	56.1

RC, record count; C, number of citation; AC, average citation per document.

**Table 2.** Top 20 Most Cited Articles According to Total Citations on Gastrointestinal Bleeding

**No**	**Article**	**Author**	**Journal**	**PY**	**TC**	**AC**
1	Risk assessment after acute upper gastrointestinal haemorrhage	Rockall et al^17^	*GUT*	1996	859	33.04
2	Transfusion strategies for acute upper gastrointestinal bleeding	Villanueva et al^18^	*New England Journal of Medicine*	2013	831	92.33
3	Risk of upper gastrointestinal-bleeding and perforation associated with individual nonsteroidal antiinflammatory drugs	Rodriguez et al^19^	*Lancet*	1994	798	28.5
4	Outcome of patients with obscure gastrointestinal bleeding after capsule endoscopy: report of 100 consecutive cases	Pennazio et al^20^	*Gastroenterology*	2004	665	36.94
5	Incidence of and mortality from acute upper gastrointestinal hemorrhage in the united-kingdom	Rockall et al^21^	*British Medical Journal*	1995	640	23.7
6	Risk-factors for gastrointestinal-bleeding in critically ill patients	Cook et al^22^	*New England Journal of Medicine*	1994	640	22.86
7	Endoscopic therapy for acute nonvariceal upper gastrointestinal hemorrhage - a metaanalysis	Cook et al^23^	*Gastroenterology*	1992	578	19.27
8	A risk score to predict need for treatment for upper-gastrointestinal haemorrhage	Blatchford et al^24^	*Lancet*	2000	524	23.82
9	The first prospective controlled trial comparing wireless capsule endoscopy with push enteroscopy in chronic gastrointestinal bleeding	Ell et al^25^	*Endoscopy*	2002	509	25.45
10	Risk of gastrointestinal haemorrhage with long term use of aspirin: meta-analysis	Derry et al^26^	*BMJ-British Medical Journal*	2000	488	22.18
11	Antibiotic prophylaxis for the prevention of bacterial infections in cirrhotic patients with gastrointestinal bleeding: a meta-analysis	Bernard et al^27^	*Hepatology*	1999	485	21.09
12	A comparison of sucralfate and ranitidine for the prevention of upper gastrointestinal bleeding in patients requiring mechanical ventilation	Cook et al^28^	*New England Journal of Medicine*	1998	472	19.67
13	Epidemiology and outcome of patients hospitalized with acute lower gastrointestinal hemorrhage: a population-based study	Longstreth^29^	*American Journal of Gastroenterology*	1997	470	18.8
14	Propranolol for prevention of recurrent gastrointestinal-bleeding in patients with cirrhosis - a controlled-study	Lebrec et al^30^	*New England Journal of Medicine*	1981	469	11.44
15	Preventing recurrent upper gastrointestinal bleeding in patients with helicobacter pylori infection who are taking low-dose aspirin or naproxen	Chan et al^31^	*New England Journal of Medicine*	2001	455	21.67
16	A meta-analysis of the yield of capsule endoscopy compared to other diagnostic modalities in patients with obscure gastrointestinal bleeding	Triester et al^32^	*American Journal of Gastroenterology*	2005	435	25.59
17	Epidemiology of hospitalization for acute upper gastrointestinal hemorrhage - a population-based study	Longstreth^33^	*American Journal of Gastroenterology*	1995	407	15.07
18	Portal-hypertension, size of esophageal-varices, and risk of gastrointestinal bleeding in alcoholic cirrhosis	Lebrec et al^34^	*Gastroenterology*	1980	386	9.19
19	Consensus recommendations for managing patients with nonvariceal upper gastrointestinal bleeding	Barkun et al^35^	*Annals of Internal Medicine*	2003	377	19.84
20	Acute upper GI bleeding: did anything change? Time trend analysis of incidence and outcome of acute upper GI bleeding between 1993/1994 and 2000	Van Leerdam et al^36^	*American Journal of Gastroenterology*	2003	376	19.79

PY, publication year; TC, total citation; AC, average citations per year.

**Table 3.** The Most Frequently Used 92 Keywords on Gastrointestinal Bleeding

**Keywords**	**Number of Uses**	**Keywords**	**Number of Uses**	**Keywords**	**Number of Uses**
Gastrointestinal bleeding	678	GI bleeding	31	Dieulafoy’s lesion	20
Upper gastrointestinal bleeding	311	Gastrointestinal bleed	30	Peptic ulcer disease	20
Gastrointestinal hemorrhage	279	Hematemesis	30	Anticoagulation	19
Endoscopy	248	Portal hypertension	29	Blood transfusion	19
Mortality	130	Small bowel	29	Clopidogrel	19
Capsule endoscopy	125	Enteroscopy	28	Computed tomography	19
Obscure gastrointestinal bleeding	120	Helicobacter pylori	28	Double-balloon enteroscopy	19
Hemorrhage	108	Nonvariceal upper gastrointestinal bleeding	28	Octreotide	19
Bleeding	107	Gastrointestinal hemorrhage	27	Upper gastrointestinal bleed	19
Lower gastrointestinal bleeding	86	Emergency department	26	Video capsule endoscopy	19
Angiography	76	Endoscopic hemostasis	26	Colon	18
Embolization	73	Haemorrhage	26	Duodenum	18
Angiodysplasia	60	Pseudoaneurysm	26	Gastrointestinal tract, hemorrhage	18
Peptic ulcer	60	Variceal bleeding	26	Left ventricular assist device	18
Outcome (s)	58	Varices	26	Acute gastrointestinal bleeding	17
Gastrointestinal haemorrhage	57	Stomach	25	Diagnostic yield	17
Colonoscopy	56	Diagnosis	24	Gastrointestinal endoscopy	17
Aspirin	51	Rockall score	24	Non-variceal upper gastrointestinal bleeding	17
Cirrhosis	51	Warfarin	24	Treatment	17
Proton pump inhibitor (s)	49	Case report	23	Complications	16
Risk factors	47	Elderly	23	Esophageal varices	16
Gastrointestinal	46	Ulcer	23	Gastric ulcer	16
Rebleeding	41	Duodenal ulcer	22	Heart failure	16
Prognosis	40	Epidemiology	22	Meckel’s diverticulum	16
Surgery	38	Hematochezia	22	Nonsteroidal anti-inflammatory drugs	16
Melena	37	Liver cirrhosis	22	Upper GI bleeding	16
NSAID (s)	34	Anticoagulants	21	Etiology	15
Small intestine	33	Endoscopic therapy	21	Glasgow-blatchford score	15
Hemostasis	32	Risk assessment	21	Non-steroidal anti-inflammatory drugs	15
Upper gastrointestinal hemorrhage	32	Acute upper gastrointestinal bleeding	20	Transfusion	15
Anemia	31	Atrial fibrillation	20		
